# Endoscopic Versus Surgical Therapy for Early Esophagogastric Junction Adenocarcinoma Based on Lymph Node Metastasis Risk: A Population-Based Analysis

**DOI:** 10.3389/fonc.2021.716470

**Published:** 2021-12-17

**Authors:** Hua Ye, Ping Chen, Yi-Fan Wang, Xiu-Jun Cai

**Affiliations:** ^1^ Department of General Surgery, Sir Run Run Shaw Hospital, Zhejiang University School of Medicine, Hangzhou, China; ^2^ Department of Gastrointestinal and Hernia Ward, HwaMei Hospital, University Of Chinese Academy Of Sciences, Ningbo, China; ^3^ Key Laboratory of Diagnosis and Treatment of Digestive System Tumors of Zhejiang Province, Ningbo, China

**Keywords:** endoscopic treatment, surgery, esophagogastric junction adenocarcinoma, lymph node metastasis, survival

## Abstract

**Background:**

In this study, we aimed to compare the prognosis and lymph node metastasis (LNM) risk in patients with early-stage esophagogastric junction (EGJ) adenocarcinoma after endoscopic treatment (ET) or radical surgery.

**Methods:**

We collected data from eligible patients based on the Surveillance, Epidemiology, and End Results (SEER) database between 2004 and 2016. Logistic regression analysis was used to determine independent predictors of LNM (examination of at least 16 lymph nodes). Cox regression analysis and propensity score-matched (PSM) analysis were subsequently utilized to compare the overall survival (OS) and cancer-specific survival (CSS) of patients treated with ET or radical surgery.

**Results:**

In total, 3708 patients were identified. Among them, 856 patients had greater than or equal to 16 examined lymph nodes (LNs) (LNE≥16). The LNM rates were 18.8% in all patients 8.3% in T1a patients and 24.6% in T1b patients. Independent predictors of LNM were submucosal invasion, tumor size ≥3cm and decreasing differentiation (P<0.05). The LNM rate decreased to approximately 5.3% in T1b tumors with well differentiation and tumor size <3cm. However, the LNM incidence increased to 17.9% or 33.3% in T1a tumors with poor differentiation or with both tumor size≥3cm and poor differentiation. Cox regression analysis demonstrated CSS was not significantly different in early-stage EGJ adenocarcinoma patients undergoing ET and those treated with radical surgery (HR= 1.004, P=0.974), which were robustly validated after PSM analysis. Moreover, subgroup analysis stratified by T1a and T1b showed similar results.

**Conclusions:**

The findings of this study indicated ET as an alternative to radical surgery in early EGJ adenocarcinoma.

## Introduction

In recent years great changes have been made in the clinical intervention for early malignant and precancerous lesions of the upper gastrointestinal (GI) tract, from radical surgery to endoscopic treatment. The incidence of esophagogastric junction (EGJ) adenocarcinoma has been rapidly rising in Western countries in the last few decades (1). A similar trend has been observed in Asia, probably due to the available eradication therapy for Helicobacter pylori (H.pylori), a high prevalence of gastroesophageal reflux disease and obesity, and dietary factors (2), and partly shared with those of gastric adenocarcinoma, i.e. H.pylori infection and dietary factors (3). As a minimally invasive approach, endoscopic submucosal dissection (ESD) or endoscopic mucosal resection (EMR) is also curative for superficial GI malignancies, including esophageal, gastric, and colonic lesions (4). Moreover, due to the varied incidence of lymph node metastasis (LNM) in esophageal and gastric cancer, there are also differences in the curative resection criteria of ESD/EMR between esophageal and gastric cancer (5, 6). However, it is unknown which curative resection criteria are better for EGJ adenocarcinoma since the incidence of metastatic EGJ adenocarcinoma remains unknown. It is noteworthy that inaccessible assessment of pathologic lymph node (LN) is considered the main drawback of endoscopic treatment (ET), as it can significantly affect patients’ survival in the case of metastatic LNs. Therefore, clinical decision-making in early-stage EGJ adenocarcinoma can be optimized by better pretreatment LNM risk stratification according to both patient and tumor features.

In this study, eligible patients from the Surveillance, Epidemiology, and End Results (SEER) database were utilized to determine preoperative predictors of LNM, followed by a comparison of the effects of radical surgery and ET on long-term survival in early-stage EGJ adenocarcinoma. Finally, an early-stage EGJ adenocarcinoma therapeutic algorithm was proposed for patients at acceptable risk for ET.

## Materials and Methods

### Origins of Materials

The National Cancer Institute (NCI) supports the SEER database, which records data on tumor incidence and survival by covering almost 28% of the population in the USA from diverse geographic regions (18 cancer registries) from 2004 to 2016. The collection and recoding of SEER data were performed using data items and codes based on the North American Association of Central Cancer Registries (NAACCR) (7). Access to the SEER database was obtained, and our study gained institutional approval.

### Inclusion and Exclusion Criteria

In total, 3708 patients were enrolled. The inclusion criteria were as follows: (1) year of diagnosis (from 2004 to 2016); (2) patients were 18 years or older; (3) histological type included adenocarcinoma (8140), mucinous adenocarcinoma (MAC) (8480), and signet ring cell cancer (SRCC) (8490); (4) available active follow-up data; and (5) patients with T1 EGJ adenocarcinoma (site codes, C15.5, C16.0, C16.1, and C16.2) treated with either ET, radical surgery According to the records in the SEER database, ET referred to endoscopic treatment for local tumor excision with pathology specimen. In addition, the definition of radical surgery was all forms of partial esophagus removal along with partial or total gastrectomy (6). At least 16 regional lymph nodes (LNs) were examined after surgical resection. The exclusion criteria were as follows: (1) distant metastasis; (2) patients who received neoadjuvant therapy; (3) patients who had more than one primary malignancy, except those with EGJ as the first diagnosis; (4) patients who died within 1 month, which was mostly caused by surgical complications; and (5) patients undergoing local tumor destruction without a pathological specimen.

There are controversies over the staging classification system for esophagogastric junction adenocarcinoma. The cancers involving it with epicenters no more than 2cm into the gastric cardia are staged as adenocarcinomas of the esophagus and those with more than 2cm involvement of the gastric cardia are staged as gastric cancers (8). Studies have shown that patients with ≥ 16 pathologically examined LNs (eLNs) have better prognoses as compared to those with < 16 eLNs (9). The American Joint Committee on Cancer (AJCC) advocates for the retrieval of at least 16 LNs for optimizing the radicality of D2 lymphadenectomies and enabling proper staging of gastric cancer (10). Therefore, we selected patients with radical surgical resection and dissection of at least 16 lymph nodes for further analysis of LNM risks in patients with early-stage EGJ adenocarcinoma.

### Statistical Analysis

Age at diagnosis, race, year of diagnosis, marital status, gender, tumor size, differentiation grade, survival (months), number of examined LNs, LNM, histology, and death cause were collected from the SEER database. The main endpoints included overall survival (OS) and cancer-specific survival (CSS).

For comparisons among groups, categorical variables were analyzed by Fisher’s exact test or Pearson’s test. Risk factors for LNM were determined by both univariate and multivariate logistic regression models, shown as odds ratios (ORs) along with 95% confidence intervals (CIs). Moreover, adjusted hazard ratios (HRs) along with 95% CIs were calculated by both univariate and multivariate Cox regression models. Additionally, PSM analysis was performed by using the 1:1 “nearest neighbor” match paradigm, aiming at further adjustment of variations in general data and bias minimization. The following covariates histology, grade, race, gender, age, T stage, tumor size, year of diagnosis, and marital status were used in PSM analysis. After matching, we compared two groups with control for covariate balance and similarity in baseline covariates between groups, and two matched groups were compared according to the study objectives. Statistical analysis was performed by R software version R-3.6.2 (The R Foundation for Statistical Computing, Vienna, Austria) as well as SPSS version 23.0 (SPSS Inc., Chicago, IL, USA). GraphPad Prism 6.0 (GraphPad Software, San Diego, CA) was employed to plot survival curves. A two-sided P value < 0.05 suggested statistical significance.

## Results

### Patient Characteristics

In total, 3708 eligible patients were included (surgical therapy: n = 2418, 65.2%; ET: n = 1290, 34.8%). Among them, 3708 patients were male and the remaining 610 were female. The median age at diagnosis was 67 years, ranging from 22 to 97 years (mean ± SD:66.35 ± 10.61 years). The median follow-up was 44 months, ranging between 1 and 155 months. In total, 1610 patients had radical surgery of partial esophagus removal along with partial gastrectomy and 808 had the radical surgery of partial esophagus removal along with total gastrectomy. Detailed data on patient demographics as well as tumor characteristics are shown in **Table 1**.

**Table 1:** Baseline characteristics of patients treated with ES and ET for early-stage esophageal cancer before and after the propensity score-matched (1:1 matching).

Characteristic	Before matched		Statistic	p	After matched		Statistic	p
	Surgery	ET			Surgery	ET		
	N=2418,%	N=1290,%			N=920,%	N=920,%		
**Gender**			χ^2 ^= 4.104	0.043			χ^2 ^= 0.434	0.510
Female	376 (15.6)	234 (18.1)			166 (18.0)	177 (19.2)		
Male	2042 (84.4)	1056 (81.9)			754 (82.0)	743 (80.8)		
**Age (years)**			χ^2 ^= 190.802	<0.001			χ^2 ^= 5.161	0.160
Up to 49	170 (7.0)	39 (3.0)			36 (3.9)	37 (4.0)		
50-64	985 (40.7)	360 (27.9)			304 (33.0)	297 (32.3)		
65-79	1114 (46.1)	650 (50.4)			478 (52.0)	452 (49.1)		
80+	149 (6.2)	241 (18.7)			102 (11.1)	134 (14.6)		
**Race**			χ^2 ^= 2.270	0.321			χ^2 ^= 3.222	0.200
White	2270 (93.9)	1221 (94.7)			874 (95.0)	864 (93.9)		
Black	56 (2.3)	32 (2.5)			24 (2.6)	21 (2.3)		
Others*	92 (3.8)	37 (2.9)			22 (2.4)	35 (3.8)		
**Tumor size (cm)**			χ^2 ^= 374.707	<0.001			χ^2 ^= 4.393	0.355
<1	511 (21.1)	351 (27.2)			258 (28.0)	236 (25.7)		
1-2	571 (23.6)	211 (16.4)			196 (21.3)	178 (19.3)		
2-3	420 (17.4)	86 (6.7)			72 (7.8)	84 (9.1)		
3+	431 (17.8)	70 (5.4)			70 (7.6)	66 (7.2)		
Not stated	485 (20.1)	572 (44.3)			324 (35.2)	356 (38.7)		
**Year of diagnosis**			χ^2 ^= 337.009	<0.001			χ^2 ^= 2.772	0.428
2004-2006	577 (23.9)	116 (9.0)			113 (12.3)	104 (11.3)		
2007-2009	675 (27.9)	189 (14.7)			188 (20.4)	183 (19.9)		
2010-2012	555 (23.0)	315 (24.4)			232 (25.2)	212 (23.0)		
2013-2016	611 (25.3)	670 (51.9)			387 (42.1)	421 (45.8)		
**Marital status**			χ^2 ^= 15.807	<0.001			χ^2 ^= 5.671	0.059
Married	1687 (69.8)	819 (63.5)			609 (66.2)	560 (60.9)		
Single/widowed	402 (16.6)	270 (20.9)			173 (18.8)	203 (22.1)		
Other/unknown	329 (13.6)	201 (15.6)			138 (15.0)	157 (17.1)		
**T stage**			χ^2 ^= 400.549	<0.001			χ^2 ^= 1.844	0.398
T1a	979 (40.5)	927 (71.9)			592 (64.3)	595 (64.7)		
T1b	1226 (50.7)	226 (17.5)			235 (25.5)	217 (23.6)		
T1x	213 (8.8)	137 (10.6)			93 (10.1)	108 (11.7)		
**Grade**			χ^2 ^= 279.570	<0.001			χ^2 ^= 4.461	0.216
Well-differentiated	346 (14.3)	210 (16.3)			160 (17.4)	134 (14.6)		
Moderately differentiated	1019 (42.1)	438 (34.0)			352 (38.3)	338 (36.7)		
Poorly/Undifferentiated	726 (30.0)	191 (14.8)			167 (18.2)	182 (19.8)		
Unknown	327 (13.5)	451 (35.0)			241 (26.2)	266 (28.9)		
**Histology**			χ^2 ^= 21.284	<0.001			χ^2 ^= 0	1.0
Adenocarcinoma	2270 (93.9)	1255 (97.3)			887 (96.4)	887 (96.4)		
Mucinous carcinoma	25 (1.0)	8 (0.6)			8 (0.9)	8 (0.9)		
Signet ring cell carcinoma	123 (5.1)	27 (2.1)			25 (2.7)	25 (2.7)		

ET, Endoscopic therapy; T1a,tumor invades the lamina propria or muscularis mucosa; T1b, tumor invades the submucosa; T1x, unknown T1a or T1b.*American Indian/Alaska Native, Asian/Pacific Islander.

### LNM Risks in Early-Stage EGJ Adenocarcinoma

In total, we collected information from 856 patients with EGJ adenocarcinoma diagnosed between 2004 and 2016 with at least 16 LNs examined who received surgical resection. The overall LNM rate was 18.8% (161/856). When stratified by pT stage, LNM rates were 8.3% (25/300) and 24.6% (122/496) in T1a and T1b patients, respectively. LNM rate decreased to 5.3% (2/38) in well-differentiated T1b tumors with a tumor size<3cm; while LNM incidence increased to 17.9% (12/67) in poorly-differentiated T1a tumors, and rose to as high as 33.3% (5/15) in poorly-differentiated tumors exceeding 3cm in size. Given that the tumor size is a key determinant of LNM, 722 patients with known tumor sizes were selected for further univariate and multivariate logistic regression analyses to identify risk factors for LNM. Consequently, we robustly found that tumor size, tumor grade, and pT stage were significant predictive indicators for LNM. LNM rate was significantly higher in T1b than T1a tumors (OR: 2.168, 95% CI: 1.273-3.692, P=0.004). Compared with small tumors that were less than 1cm in size, the risk of LNM was increased in tumor sizes exceeding 3 cm (OR=5.484, 95% CI: 2.688-11.187, P <0.001) in multivariate analysis. The incidence of LNM was also significantly higher in tumors with poor/moderate differentiation or undifferentiation than those with well differentiation (OR 2.824, 95% CI: 1.071-7.443, P=0.036; OR 4.783, 95% CI 1.812-12.624, P= 0.002, respectively) in multivariate analysis. The detailed patient characteristics are summarized in **Table 2**. According to the present NCCN guidelines, ET is recommended for T1a tumors but is less definitive for T1b tumors.

**Table 2:** Logistic regression analysis of the risk factors for lymph node metastasis in early-stage esophagogastric junction cancer (LNE≥16).

Characteristic	Univariate analysis		Multivariate analysis	
	OR (95% CI)	*P*	OR (95% CI)	*P*
**Gender**				
Female	Reference			
Male	1.216 (0.762-1.942)	0.412		
**Age (years)**				
Up to 49	Reference			
50-64	0.904 (0.455-1.794)	0.773		
65-79	0.946 (0.480-1.865)	0.872		
80+	1.910 (0.737-4.948)	0.183		
**Race**				
White	Reference			
Black	0.236 (0.031-1.785)	0.162		
Others*	1.135 (0.510-2.525)	0.756		
**Tumor size (cm)**				
<1	Reference		Reference	
1-2	2.556 (1.256-5.201)	0.010	1.699 (0.813-3.554)	0.159
2-3	3.403 (1.638-7.070)	0.001	1.930 (0.896-4.156)	0.093
3+	8.868 (4.496-17.490)	<0.001	5.524 (2.716-11.234)	<0.001
Not stated	1.350 (0.576-3.166)	0.490	1.130 (0.466-2.738)	0.787
**pT stage**				
T1a	Reference		Reference	
T1b	3.588 (2.271-5.670)	<0.001	2.162 (1.311-3.565)	0.003
T1x	3.348 (1.622-6.912)	0.001	2.729 (1.234-6.035)	0.013
**Year of diagnosis**				
2004-2006	Reference			
2007-2009	1.410 (0.830-2.397)	0.204		
2010-2012	1.174 (0.690-1.998)	0.553		
2013-2016	0.986 (0.586-1.661)	0.959		
**Marital status**				
Married	Reference			
Single/widowed	1.258 (0.789-2.006)	0.335		
Other/unknown	0.881 (0.517-1.501)	0.640		
**Grade**				
Well-differentiated	Reference		Reference	
Moderately differentiated	3.614 (1.518-8.602)	0.004	2.539 (1.042-6.186)	0.040
Poorly/Undifferentiated	7.558 (3.202-17.840)	<0.001	4.325 (1.774-10.544)	0.001
Unknown	1.158 (0.341-3.932)	0.814	1.275 (0.358-4.533)	0.708
**Histology**				
Adenocarcinoma	Reference		Reference	
Mucinous carcinoma	1.332 (0.274-6.480)	0.723	0.611 (0.115-3.253)	0.563
Signet ring cell carcinoma	2.331 (1.322-4.110)	0.003	1.798 (0.965-3.350)	0.065

LNE, Number of examined lymph nodes; OR, odd ratio; 95% CI,95% confidence intervals; pT, pathologic tumor; T1a,tumor invades the lamina propria or muscularis mucosa; T1b, tumor invades the submucosa.

*American Indian/Alaska Native, Asian/Pacific Islander.

### LNM Rates in T1a Tumors

The rate of LNM in T1a tumor sizes exceeding 3 cm was 23.8% (10/42) compared with 6.1% (12/197) in tumors <3 cm in size. Compared with small tumors less than 1cm in size, the risk of LNM was increased in tumor sizes exceeding 3 cm (OR=4.662, 95% CI: 1.407-15.442, P =0.012) in multivariate analysis. The presence of LNM was 4.8% (3/62), 7.0% (8/115), and 17.9% (12/67) in well-differentiated, moderately differentiated, and poorly/undifferentiated T1a tumors, respectively. The incidence of LNM was higher in poorly differentiated T1a cancer compared with well-differentiated examples (OR3.611,95% CI: 0.865-15.085, P =0.078) in multivariate analysis. The details of other tumor features is shown in **Table 3**.

**Table 3:** Logistic regression analysis of the risk factors for lymph node metastasis in T1a and T1b esophagogastric junction cancer (LNE≥16).

Characteristic	T1a				T1b			
	Univariate analysis		Multivariate analysis		Univariate analysis		Multivariate analysis		
	OR (95% CI)	*P*	OR (95% CI)	*P*	OR (95% CI)	*P*	OR (95% CI)	*P*
**Gender**								
Female	Reference				Reference			
Male	1.710 (0.493-5.930)	0.398			1.216 (0.706-2.095)	0.481		
**Age (years)**								
Up to 49	Reference				Reference			
50-64	0.687 (0.207-2.276)	0.539			1.029 (0.390-2.716)	0.954		
65-79	0.433 (0.121-1.548)	0.198			1.076 (0.413-2.801)	0.881		
80+	Omitted				1.875 (0.551-6.379)	0.314		
**Race**								
White	Reference				Reference			
Black	Omitted				1.053 (0.410-2.699)	0.915		
Others*	1.067 (0.131-8.691)	0.952			0.352 (0.037-3.374)	0.365		
**Tumor size (cm)**								
<1	Reference		Reference		Reference		Reference	
1-2	1.516 (0.422-5.446)	0.524	1.342 (0.364-4.943)	0.658	2.410 (0.882-6.587)	0.086	2.036 (0.732-5.666)	0.173
2-3	1.617 (0.295-8.846)	0.580	1.126 (0.191-6.633)	0.896	2.686 (0.969-7.447)	0.058	2.292 (0.809-6.490)	0.118
3+	6.062 (1.928-19.060)	0.002	4.673 (1.421-15.371)	0.011	7.019 (2.622-18.791)	<0.001	6.091 (2.239-16.570)	<0.001
Not stated	1.003 (0.231-4.355)	0.996	0.984 (0.219-4.423)	0.983	1.068 (0.289-3.943)	0.921	1.042 (0.277-3.921)	0.951
**Year of diagnosis**								
2004-2006	Reference				Reference			
2007-2009	0.486 (0.128-1.851)	0.290			1.611 (0.850-3.053)	0.144		
2010-2012	0.736 (0.254-2.132)	0.573			1.239 (0.655-2.344)	0.511		
2013-2016	0.623 (0.216-1.796)	0.381			0.974 (0.526-1.806)	0.934		
**Marital status**								
Married	Reference				Reference		Reference	
Single/widowed	0.597 (0.170-2.092)	0.420			1.759 (1.012-3.055)	0.045	1.780 (0.981-3.232)	0.058
Other/unknown	0.531 (0.119-2.370)	0.406			0.888 (0.469-1.681)	0.715	0.879 (0.448-1.724)	0.707
**Grade**								
Well-differentiated	Reference		Reference		Reference		Reference	
Moderately differentiated	1.470 (0.376-5.754)	0.580	1.543 (0.380-6.259)	0.544	3.985 (1.184-13.404)	0.026	3.005 (0.872-10.359)	0.081
Poorly/Undifferentiated	4.291 (1.149-16.021)	0.030	3.909 (0.973-15.708)	0.055	7.179 (2.154-23.931)	0.001	4.944 (1.440-16.970)	0.011
Unknown	0.728 (0.117-4.527)	0.734	0.820 (0.127-5.298)	0.835	2.095 (0.317-13.835)	0.442	1.496 (0.207-10.794)	0.690
**Histology**								
Adenocarcinoma	Reference		Reference		Reference		Reference	
Mucinous carcinoma	11.727 (0.709-193.969)	0.085	5.434 (0.185-160.030)	0.327	0.667 (0.077-5.776)	0.713	0.497 (0.053-4.627)	0.539
Signet ring cell carcinoma	1.466 (0.316-6.791)	0.625	0.76 (0.146-4.036)	0.755	2.578 (1.320-5.037)	0.006	2.025 (0.980-4.184)	0.057

LNE, Number of examined lymph nodes; OR, odd ratio; 95% CI,95% confidence intervals; T1a,tumor invades the lamina propria or muscularis mucosa.

*American Indian/Alaska Native, Asian/Pacific Islander.

### LNM Rates in T1b Tumors

We further compared the LNM rate in T1b tumors between tumor size exceeding 3 cm and tumors <3 cm, which was 42.7% (56/131) versus 19.3% (61/316). The incidence of LNM was higher in Signet ring cell carcinoma (OR 2.073, 95% CI: 1.006-4.273, P = 0.048) than in well-differentiated tumors. Compared with small tumors of less than 1cm in size, the risk of LNM was increased in tumor sizes exceeding 3 cm (OR=5.935, 95% CI: 2.183-16.134, P<0.001). The presence of LNM was 6.4% (3/47), 21.4% (47/220), and 32.9% (70/213) in well-differentiated, moderately differentiated, and poorly/undifferentiated T1b tumors, respectively. LNM incidence was higher in poorly-differentiated than well-differentiated T1b tumors (OR 7.287, 95%CI: 1.674-31.725, P=0.008) in multivariate analysis. The details of other tumor features are shown in **Table 3**.

### Patient Survival

**Figure 1:**
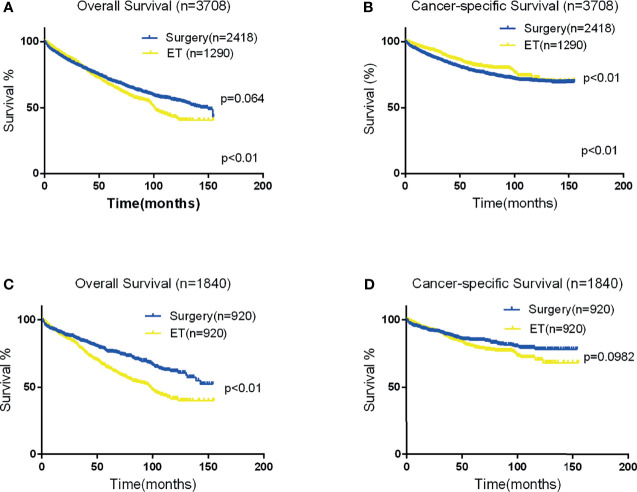
Kaplan-Meier curves for OS and CSS. Panels **(A, B)** depict the overall and CSS of the Two groups in the original data set, and panels **(C, D)** depict the OS and CSS of the two group after propensity score matching.

The mean OS in the surgical therapy and ET groups was 105 months (95% CI 103–108), 97 months (95% CI 93–102) respectively. The log-rank test showed that overall survival was similar in patients treated by surgical therapy and ET (p=0.065). Survival curves of the two groups were displayed in **Figure 1A**. The mean CSS was 121 months (95% CI 118–123) and 126 months (95% CI 122–131) in the surgical therapy, ET groups, respectively. The log-rank test revealed that the CSS survival of patients treated by surgical therapy was significantly worse than those treated by ET (P<0.001). The survival curves of the two groups are displayed in **Figure 1B**, after propensity score matching. Furthermore, The mean OS in the radical surgery of partial esophagus removal along with partial gastrectomy, total gastrectomy, and ET groups was 107 months (95% CI 103–110), 103months (95% CI 99–108), and 97 months (95% CI 93–102) respectively. A log-rank test showed that OS was similar in patients treated by the radical surgery of partial esophagus removal along with partial gastrectomy, total gastrectomy, and ET groups (p=0.081). The mean CSS in the radical surgery of partial esophagus removal along with partial gastrectomy, total gastrectomy, and ET groups was 121 months (95% CI 118–124), 120months (95% CI 116–124), and 127 months (95% CI 122–131) respectively. The log-rank test revealed that the CSS survival of patients treated by ET was significantly better than those treated by surgery of partial gastrectomy group (P=0.002) and those treated by surgery of total gastrectomy group (P=0.001).The multivariate Cox regression models showed that OS (ET: HR 1.220, 95% CI: 1.059-1.406, P =0.006) and CSS (ET: HR1.004, 95% CI: 0.807-1.249, P=0.974.) compare with the surgical therapy group. Moreover, univariate and multivariate Cox regression models consistently revealed that tumor size(≥2cm), year of diagnosis, pT stage, LNM, Grade(Poorly/Undifferentiated), histology (Signet ring cell carcinoma), marital status, and old age (≥65years) were significant prognostic indicators for both OS and CSS (**Table 4**).

**Table 4:** Cox regression analysis of OS and CSS in patients with early-stage esophagogastric junction cancer.

Characteristic	OS					CSS			
	Univariate analysis		Multivariate analysis			Univariate analysis		Multivariate analysis	
	HR (95% CI)	*P*	HR (95% CI)	*P*		HR (95% CI)	*P*	HR (95% CI)	*P*
**Gender**									
Female	Reference					Reference			
Male	0.998 (0.896-1.112)	0.975				0.968 (0.855-1.097)	0.612		
**Race**									
White	Reference		Reference			Reference		Reference	
Black	1.353 (1.084-1.689)	0.007	1.187 (0.949-1.484)	0.133		1.351 (1.044-1.748)	0.022	1.104 (0.851-1.431)	0.457
Others*	0.830 (0.656-1.049)	0.119	0.818 (0.646-1.036)	0.095		0.918 (0.705-1.195)	0.523	0.908 (0.696-1.185)	0.477
**Tumor size (cm)**									
<1	Reference		Reference			Reference		Reference	
1-2	1.507 (1.257-1.807)	<0.001	1.144 (0.952-1.375)	0.152		1.879 (1.474-2.394)	<0.001	1.284 (1.004-1.641)	0.046
2-3	2.115 (1.762-2.538)	<0.001	1.309 (1.084-1.579)	0.005		2.778 (2.183-3.537)	<0.001	1.469 (1.146-1.882)	0.002
3+	4.139 (3.531-4.851)	<0.001	1.564 (1.317-1.856)	<0.001		6.456 (5.224-7.979)	<0.001	1.906 (1.521-2.390)	<0.001
Not stated	2.943 (2.525-3.430)	<0.001	1.389 (1.180-1.635)	<0.001		4.176 (3.392-5.141)	<0.001	1.682 (1.349-2.096)	<0.001
**Year of diagnosis**									
2004-2006	Reference		Reference			Reference		Reference	
2007-2009	0.868 (0.784-0.962)	0.007	0.900 (0.812-0.997)	0.044		0.887 (0.786-1.001)	0.051	0.942 (0.834-1.063)	0.333
2010-2012	0.690 (0.616-0.773)	<0.001	0.741 (0.660-0.831)	<0.001		0.697 (0.611-0.795)	<0.001	0.793 (0.694-0.907)	0.001
2013-2016	0.659 (0.582-0.747)	<0.001	0.770 (0.677-0.875)	<0.001		0.630 (0.545-0.727)	<0.001	0.807 (0.697-0.935)	0.004
**Marital status**									
Married	Reference		Reference			Reference		Reference	
Single/widowed	1.436 (1.306-1.578)	<0.001	1.191 (1.080-1.312)	<0.001		1.530 (1.371-1.707)	<0.001	1.249 (1.116-1.398)	<0.001
Other/unknown	1.142 (1.016-1.284)	0.027	1.182 (1.050-1.332)	0.006		1.232 (1.077-1.409)	0.002	1.252 (1.092-1.435)	0.001
**T stage**									
T1a	Reference							Reference	
T1b	1.205 (1.083-1.340)	0.001	1.192 (1.061-1.340)	0.003		1.403 (1.229-1.601)	<0.001	1.313 (1.137-1.517)	<0.001
T1x	3.905 (3.553-4.292)	<0.001	1.443 (1.292-1.612)	<0.001		5.153 (4.594-5.779)	<0.001	1.596 (1.400-1.818)	<0.001
**Treatment**									
Surgery	Reference							Reference	
ET	1.092 (0.960-1.241)	0.180	1.220 (1.059-1.406)	0.006		0.693 (0.578-0.831)	<0.001	0.830 (0.682-1.010)	0.062
RT	6.111 (5.573-6.702)	<0.001	3.700 (3.271-4.185)	<0.001		7.031 (6.311-7.834)	<0.001	4.024 (3.483-4.649)	<0.001
**LNM**									
No	Reference		Reference			Reference		Reference	
Yes	2.275 (2.066-2.504)	<0.001	1.507 (1.361-1.668)	<0.001		2.728 (2.453-3.035)	<0.001	1.614 (1.443-1.805)	<0.001
**Grade**									
Well-differentiated	Reference		Reference			Reference		Reference	
Moderately differentiated	1.573 (1.349-1.834)	<0.001	1.084 (0.928-1.267)	0.310		1.780 (1.466-2.162)	<0.001	1.097 (0.900-1.336)	0.358
Poorly/Undifferentiated	2.368 (2.031-2.761)	<0.001	1.245 (1.060-1.461)	0.007		3.122 (2.577-3.783)	<0.001	1.393 (1.141-1.700)	0.001
Unknown	1.216 (1.026-1.440)	0.024	0.873 (0.734-1.037)	0.121		1.325 (1.069-1.642)	0.010	0.898 (0.722-1.116)	0.332
**Histology**									
Adenocarcinoma	Reference		Reference			Reference		Reference	
Mucinous carcinoma	2.136 (1.574-2.899)	<0.001	1.792 (1.319-2.435)	<0.001		2.262 (1.602-3.194)	<0.001	1.796 (1.270-2.540)	0.001
Signet ring cell carcinoma	1.779 (1.531-2.068)	<0.001	1.191 (1.018-1.393)	0.029		1.960 (1.657-2.319)	<0.001	1.184 (0.994-1.410)	0.059
**Age (years)**									
Up to 49	Reference		Reference					Reference	
50-64	1.192 (0.943-1.505)	0.141	1.161 (0.918-1.467)	0.213		1.038 (0.803-1.343)	0.774	1.006 (0.776-1.303)	0.966
65-79	1.908 (1.521-2.394)	<0.001	1.659 (1.320-2.085)	<0.001		1.514 (1.180-1.943)	0.001	1.298 (1.009-1.670)	0.043
80+	4.358 (3.452-5.502)	<0.001	2.447 (1.929-3.106)	<0.001		3.669 (2.841-4.737)	<0.001	1.937 (1.491-2.518)	<0.001

ET, Endoscopic therapy; RT, Radiotherapy; LNM, lymph node metastasis; HR, Hazard ratio; 95% CI,95% confidence intervals; T1a,tumor invades the lamina propria or muscularis mucosa; T1b, tumor invades the submucosa; T1x, unknown T1a or T1b.

*American Indian/Alaska Native, Asian/Pacific Islander.

### PSM

In total, 920 patient pairs were included in the PSM analysis. Patient features and tumor characteristics of both surgical therapy and ET groups after propensity matching were displayed in **Table 1**. As a result, all matched variables were balanced between two groups (all P > 0.05). Survival analysis and log-rank test revealed worse OS in the ET group than surgical therapy group (**Figure 1C**). There was no significant difference in CSS (**Figure 1D**). Moreover, Cox proportional hazard regression revealed significant differences in OS (HR = 1.488, 95% CI 1.240-1.786; P < 0.001) and no significant differences in CSS (HR = 1.112, 95% CI:0.866-1.429; P = 0.405) between surgical therapy and ET groups. The details of other tumor features are shown in **Table 5**.

**Table 5:** Cox regression analysis of OS and CSS in patients with early-stage esophagogastric junction cancer after propensity score matching.

Characteristic	OS					CSS			
	Univariate analysis		Multivariate analysis			Univariate analysis		Multivariate analysis	
	HR (95% CI)	*P*	HR (95% CI)	*P*		HR (95% CI)	*P*	HR (95% CI)	*P*
**Gender**									
Female	Reference					Reference			
Male	1.029 (0.819-1.293)	0.807				1.031 (0.751-1.414)	0.851		
**Race**									
White	Reference					Reference			
Black	1.182 (0.667-2.098)	0.567				0.720 (0.268-1.934)	0.515		
Others*	0.901 (0.508-1.598)	0.722				0.693 (0.286-1.680)	0.417		
**Tumor size (cm)**									
<1	Reference		Reference			Reference		Reference	
1-2	1.369 (1.028-1.822)	0.031	1.115 (0.833-1.491)	0.465		1.360 (0.908-2.038)	0.136	1.046 (0.692-1.581)	0.831
2-3	1.685 (1.190-2.386)	0.003	1.159 (0.811-1.657)	0.418		2.029 (1.277-3.223)	0.003	1.324 (0.822-2.134)	0.248
3+	2.157 (1.507-3.087)	<0.001	1.489 (1.029-2.153)	0.035		2.658 (1.664-4.245)	<0.001	1.652 (1.016-2.687)	0.043
Not stated	1.318 (1.033-1.681)	0.026	1.085 (0.841-1.400)	0.529		1.385 (0.982-1.955)	0.063	1.126 (0.785-1.614)	0.520
**Year of diagnosis**									
2004-2006	Reference		Reference			Reference		Reference	
2007-2009	0.780 (0.614-0.990)	0.041	0.733 (0.575-0.933)	0.012		0.785 (0.561-1.099)	0.158	0.705 (0.502-0.992)	0.045
2010-2012	0.578 (0.443-0.755)	<0.001	0.564 (0.428-0.742)	<0.001		0.560 (0.389-0.806)	0.002	0.517 (0.355-0.752)	0.001
2013-2016	0.615 (0.459-0.823)	0.001	0.558 (0.412-0.756)	<0.001		0.560 (0.379-0.827)	0.004	0.476 (0.317-0.714)	<0.001
**Marital status**									
Married	Reference					Reference			
Single/widowed	1.219 (0.986-1.508)	0.068				1.226 (0.914-1.645)	0.174		
Other/unknown	0.849 (0.644-1.119)	0.245				0.852 (0.583-1.244)	0.406		
**T stage**									
T1a	Reference		Reference			Reference		Reference	
T1b	1.920 (1.567-2.351)	<0.001	1.494 (1.203-1.857)	<0.001		2.310 (1.755-3.041)	<0.001	1.705 (1.270-2.289)	<0.001
T1x	2.082 (1.618-2.680)	<0.001	1.784 (1.374-2.316)	<0.001		2.499 (1.778-3.512)	<0.001	2.087 (1.464-2.976)	<0.001
**Treatment**									
ES	Reference		Reference			Reference		Reference	
ET	1.599 (1.337-1.913)	<0.001	1.488 (1.240-1.786)	<0.001		1.229 (0.962-1.570)	0.099	1.112 (0.866-1.429)	0.405
**Grade**									
Well-differentiated	Reference		Reference			Reference		Reference	
Moderately differentiated	1.246 (0.941-1.649)	0.124	1.148 (0.875-1.506)	0.822		1.251 (0.851-1.839)	0.254	1.025 (0.693-1.514)	0.903
Poorly/Undifferentiated	1.668 (1.233-2.258)	0.001	1.196 (0.874-1.636)	0.264		1.937 (1.292-2.903)	0.001	1.323 (0.868-2.017)	0.193
Unknown	0.788 (0.584-1.063)	0.118	0.752 (0.554-1.021)	0.067		0.664 (0.432-1.021)	0.062	0.621 (0.401-0.962)	0.033
**Histology**									
Adenocarcinoma	Reference		Reference			Reference		Reference	
Mucinous carcinoma	1.855 (0.829-4.152)	0.133	1.116 (0.488-2.550)	0.795		3.031 (1.249-7.353)	0.014	1.810 (0.725-4.517)	0.204
Signet ring cell carcinoma	2.042 (1.304-3.199)	0.002	1.297 (0.808-2.082)	0.281		2.413 (1.379-4.220)	0.002	1.373 (0.759-2.486)	0.295
**Age (years)**									
Up to 49	Reference		Reference			Reference		Reference	
50-64	0.982 (0.526-1.834)	0.956	1.024 (0.547-1.916)	0.941		0.559 (0.283-1.105)	0.094	0.574 (0.289-1.141)	0.113
65-79	1.796 (0.982-3.286)	0.057	1.685 (0.918-3.095)	0.092		0.980 (0.514-1.868)	0.952	0.881 (0.459-1.692)	0.704
80+	4.969 (2.687-9.188)	<0.001	3.821 (2.051-7.118)	<0.001		3.078 (1.593-5.948)	0.001	2.158 (1.102-4.226)	0.025

ET, Endoscopic therapy; HR, Hazard ratio; 95% CI,95% confidence intervals; T1a,tumor invades the lamina propria or muscularis mucosa; T1b, tumor invades the submucosa; T1x, unknown T1a or T1b.

*American Indian/Alaska Native, Asian/Pacific Islander.

### Subgroup Analysis

The 920 patient pairs were further categorized into T1a and T1b groups. After adjustment of both patient demographics and tumor variables, surgical therapy and ET related CSS (HR = 1.085, 95% CI 0.760-1.550; P = 0.653), (HR = 1.335, 95% CI: 0.856-2.083; P = 0.203) were not significantly different in T1a and T1b patients (shown in **Table 6**).

**Table 6:** Cox regression analysis of CSS in patients with T1a and T1b esophagogastric junction cancer after propensity score matching.

Characteristic	T1a					T1b			
	Univariate analysis		Multivariate analysis			Univariate analysis		Multivariate analysis	
	HR (95% CI)	*P*	HR (95% CI)	*P*		HR (95% CI)	*P*	HR (95% CI)	*P*
**Gender**									
Female	Reference					Reference			
Male	1.005 (0.640-1.579)	0.982				1.252 (0.706-2.219)	0.442		
**Race**									
White	Reference					Reference			
Black	Omitted					0.697 (0.097-5.011)	0.720		
Others*	1.310 (0.536-3.205)	0.554				Omitted			
**Tumor size (cm)**									
<1	Reference		Reference			Reference		Reference	
1-2	1.126 (0.642-1.975)	0.680	1.038 (0.589-1.829)	0.897		1.380 (0.693-2.748)	0.359	1.495 (0.721-3.100)	0.280
2-3	1.815 (0.912-3.612)	0.089	1.546 (0.767-3.115)	0.223		1.553 (0.733-3.292)	0.250	1.638 (0.754-3.559)	0.212
3+	2.167 (1.062-4.425)	0.034	2.184 (1.056-4.517)	0.035		2.133 (0.958-4.749)	0.064	1.503 (0.637-3.545)	0.352
Not stated	1.234 (0.792-1.921)	0.353	1.248 (0.788-1.976)	0.346		1.039 (0.510-2.119)	0.915	1.055 (0.498-2.233)	0.889
**Year of diagnosis**									
2004-2006	Reference		Reference			Reference			
2007-2009	0.781 (0.494-1.234)	0.290	0.835 (0.525-1.330)	0.448		1.208 (0.577-2.527)	0.617		
2010-2012	0.611 (0.369-1.011)	0.055	0.615 (0.363-1.042)	0.070		0.789 (0.373-1.670)	0.536		
2013-2016	0.458 (0.249-0.842)	0.012	0.449 (0.238-0.848)	0.014		0.948 (0.448-2.006)	0.888		
**Marital status**									
Married	Reference					Reference			
Single/widowed	1.171 (0.767-1.790)	0.465				1.446 (0.897-2.331)	0.130		
Other/unknown	0.916 (0.542-1.546)	0.741				0.798 (0.379-1.678)	0.551		
**Treatment**									
surgery	Reference		Reference			Reference		Reference	
ET	1.083 (0.764-1.536)	0.654	1.085 (0.760-1.550)	0.653		1.341 (0.877-2.049)	0.175	1.335 (0.856-2.083)	0.203
**Grade**									
Well-differentiated	Reference		Reference			Reference		Reference	
Moderately differentiated	1.059 (0.636-1.764)	0.824	0.972 (0.580-1.629)	0.915		1.479 (0.657-3.331)	0.344	1.246 (0.543-2.863)	0.604
Poorly/Undifferentiated	1.505 (0.848-2.669)	0.162	1.117 (0.614-2.031)	0.717		2.364 (1.043-5.357)	0.039	2.053 (0.882-4.776)	0.095
Unknown	0.627 (0.367-1.072)	0.088	0.536 (0.309-0.929)	0.026		1.909 (0.751-4.851)	0.175	1.936 (0.749-5.005)	0.173
**Histology**									
Adenocarcinoma	Reference		Reference			Reference		Reference	
Mucinous carcinoma	3.494 (0.863-14.140)	0.079	4.054 (0.989-16.618)	0.052		2.108 (0.517-8.601)	0.299	2.476 (0.587-10.455)	0.217
Signet ring cell carcinoma	2.826 (1.240-6.441)	0.013	1.876 (0.770-4.571)	0.166		1.115 (0.352-3.530)	0.853	0.619 (0.185-2.072)	0.437
**Age (years)**									
Up to 49	Reference		Reference			Reference		Reference	
50-64	0.858 (0.303-2.431)	0.773	0.801 (0.282-2.280)	0.678		0.199 (0.061-0.650)	0.007	0.209 (0.059-0.738)	0.015
65-79	1.270 (0.461-3.497)	0.644	1.050 (0.378-2.913)	0.926		0.422 (0.151-1.180)	0.100	0.460 (0.152-1.392)	0.169
80+	4.341 (1.537-12.257)	0.006	3.060 (1.061-8.827)	0.039		0.976 (0.343-2.780)	0.964	0.966 (0.316-2.955)	0.952

ET, Endoscopic therapy; RT, Radiotherapy; LNM, lymph node metastasis; HR, Hazard ratio; 95% CI,95% confidence intervals; T1a,tumor invades the lamina propria or muscularis mucosa; T1b, tumor invades the submucosa.

*American Indian/Alaska Native, Asian/Pacific Islander.

## Discussion

Accumulated studies have demonstrated that EGJ adenocarcinoma is a separate entity from gastric or esophageal malignancies due to unique clinicopathological characteristics and patient survival (11, 12). The majority of EGJ carcinomas are handled by surgical intervention, including esophagectomy along with total or proximal gastrectomy, which, however, greatly attenuates postoperative living quality and is accompanied by a high risk of complications. The rate of postoperative complications is reported to be 33-39% according to a systematic review (13). ESD is particularly suitable for patients with early-stage proximal gastric cancer, who, otherwise, are generally treated with total gastrectomy. If patients are managed with ESD, the whole stomach can be preserved, along with better life quality (14). Due to the unknown incidence of LNM in EGJ adenocarcinoma, there is no consensus on the indication of endoscopic resection for superficial EGJ adenocarcinoma.

To our knowledge, our study is the largest to date concerning LNM rates in early-stage EGJ adenocarcinoma after eliminating patients with less than 16 examined LNs. We found that the LNM rate in early-stage EGJ adenocarcinoma was as high as 18.8% (161/856). LNM rates stratified by pT stage were 8.3% (25/300) in T1a and 24.6% (122/496) in T1b. Moreover, the rate of LNM decreased to 5.3% (2/38) in well-differentiated T1b tumors with tumor size<3cm; and LNM rate increased to 17.9% (12/67) in poorly differentiated T1a tumors, and to 33.3% (5/15) in poorly differentiated T1a tumors with tumor size>3cm. Overall, there is limited information concerning the LNM rate in superficial EGJ adenocarcinoma. According to the study by Gertler, LNM was only detectable in pT1b tumors (18%) but not in pT1a among superficial EGJ adenocarcinoma (15), which was also similarly reported by Stein (16). Moreover, Koufuji, et al. reported no LNM in T1 EGJ carcinoma (17). Of the above studies, the relatively inadequate sample size might be the most significant drawback. Zhu, et al. reported that the overall LNM rate of superficial EGJ carcinoma was 21.75%, which is 11.41% and 26.50% in mucosal cancer and submucosal cancer, respectively. The results of the above study are consistent with our findings and another study concerning surgically resected pT1 EGJ carcinoma (18, 19).

Previous studies have shown that tumor size, pathological differentiation, lymphovascular invasion, and infiltration depth are risk factors for LNM in gastric and esophageal cancer (15, 19). In our study, similar predictors of LNM involvement were revealed, including tumor size, differentiation type, and depth of invasion. To be specific, poor tumor differentiation (including moderately/poorly differentiated and undifferentiated) and tumor sizes exceeding 3 cm increased LNM risk. Tumor differentiation is the most potent predictor. Therefore, endoscopic intervention might be proper for low-risk patients, while, high-risk patients should be managed by surgical resection in consideration of the high risk of LNM.

Our study revealed that the CSS survival of patients treated by surgical therapy was significantly worse than those treated by ET (P<0.001). Subset analysis of survival of ET vs surgery including radical surgery of partial esophagus removal along with partial gastrectomy group and total gastrectomy group. A log-rank test revealed that the CSS survival of patients treated by ET was significantly better than those treated by surgery of partial gastrectomy group (P=0.002) and those treated by surgery of total gastrectomy group (P=0.001). Better survival of the ET group in the overall population is related to the selection bias of patients with less advanced tumors than surgery groups. Previous research has revealed that age, T stage and tumor differentiation are independently correlated with poor prognosis (20–22) Due to the bias caused these parameters which can interfere with the comparison of ET and surgical therapy, multivariate Cox regression analysis and PSM were performed. ET and surgical therapy were associated with similar CSS in patients with early-stage EGJ adenocarcinoma. Additionally, subgroup analysis stratified by T stage also showed similar outcomes. PSM analysis also revealed consistent outcomes, which could decrease selection bias associated with diverse clinical features of ET and surgical therapy. We identify an OS benefit of surgery compared to ET (HR = 1.488, 95% CI:1.240-1.786; P < 0.001), but no CSS difference between surgical therapy and ET groups after PSM (HR = 1.112, 95% CI:0.866-1.429; P=0.405). Patients in the ET group may have more non-oncological basic diseases and are more likely to have non-oncological death cases. Therefore, the OS of the ET group is worse than that of the surgery group. The authors found that patients with sm1 cancers, classified by a submucosal invasion of < 500μm, and tumors smaller than 3 cm had no LNMs. Nevertheless, with a deep submucosal invasion of ≥500μm stratified by sm2 and sm3, the incidence of LNM increased to 28.6%, irrespective of tumor size. The above outcomes suggest that ESD can be safely used to treat patients with sm1 and tumor size < 3 cm, which is beyond the proposed guidelines (6, 23). Most patients with T1b tumors should be treated by surgical intervention due to the high LNM rate (24.6%). Nevertheless, LNM incidence in T1b cancer with all low-risk tumor characteristics was only 5.3%. Hence, definitive ET must be cautiously determined on submucosal cancers without other high-risk characteristics. The multivariate Cox regression models showed that no significant differences in CSS (HR = 1.004, 95% CI: 0.807-1.249, P=0.974) between surgical therapy and ET groups. Moreover, Cox proportional hazards regression revealed no significant differences in CSS (HR = 1.112, 95% CI:0.866-1.429; P = 0.405) between surgical therapy and ET groups after PSM. Therefore, ET might be a valid alternative to surgical therapy to treat early EGJ adenocarcinoma, especially in elderly patients. Marital status is not a risk factor for LNM in gastric and esophageal cancer in our study. Cox proportional hazards regression revealed that for marital status there were significant differences in OS and CSS. Divorce, widowhood, and other reasons for living alone might increase the risk of adopting bad lifestyle habits. Previous research has shown that an increased risk of esophagogastric cancers is associated with the status of being unmarried and having a low level of education and a low income (24). But after PSM Cox proportional hazards regression revealed that marital status had no significant differences in OS and CSS. The associations require attention in terms of identifying high-risk individuals.

Diagnostic ER is considered as potentially curative and also has a more accurate evaluation of invasion depth than endoscopic ultrasonography (EUS) (25), which is a feasible and reasonable final step in all early-stage EGJ adenocarcinoma. Pathologic assessment on ER samples could assist further therapeutic strategies, which should simultaneously consider patient-related parameters. Moreover, a multidisciplinary team involving surgeons, medical oncologists, and endoscopists is necessary for clinical decision-making. For patients with older age or multiple comorbidities, a higher probability of leaving positive LNs may be acceptable for a lower morbidity procedure. Conversely, aggressive surgical therapy should be considered among young patients even with low risks of LNM.

In this large population-based study, our findings are mainly based on real-world outcomes. To our knowledge, this is the first population-based study to describe the long-term survival of ET in comparison with surgery for early-stage EGJ adenocarcinoma. Nevertheless, certain limitations must be acknowledged, Firstly, relevant data on lymphovascular invasion, the deep distance of submucosal invasion, and macroscopic type are inaccessible in the SEER database, which are potential risk factors for LNM. The absence of these variables might affect the accurate assessment of LNM. Secondly, the applied models are simplified and only use available and accepted measures, which do not adequately account for all variables associated with subject outcomes. The lack of records of surgical complications in the SEER database affects results on the influence of complications and cancer survival. We excluded patients who died within one month after surgery to reduce the impact of surgical complications. Additionally, the lack of a comorbidity index may have an impact on assessing patients’ choice of treatment modality, as older patients and those with a higher Comorbidity Index had lower odds of being treated with surgery. Selection biases are unavoidable in the retrospective analysis. Therefore, to reduce bias as much as possible, we applied the PSM method to ensure that the clinical data between the ET group and the surgery group were consistent, such as age, gender, tumor size, etc. Finally, although PSM was further performed in this study, the results must be cautiously interpreted due to the fraction of unmatched patients.

## Conclusion

This population-based study reveals that LNM risk is significantly increased in submucosal compared with intramucosal tumors. In subgroup analysis, patients with poorly-differentiated T1a cancers with a size of >3 cm had an increased LNM rate than those with T1b cancers without other high-risk factors. These data suggest disease heterogeneity among patients with early-stage EGJ adenocarcinoma, which must be identified to select the optimal resection strategy. Therefore, we believe that national guidelines for the management of early-stage EGJ adenocarcinoma should include all high risk features for LNM and stage-specific surgery therapy mortality. ET is thus a valid alternative to surgery for T1a tumors and well-differentiated T1b tumors with a tumor size of <3cm in early EGJ adenocarcinoma, especially for older patients. ET is a minimally invasive surgery with less trauma and higher quality of life compared to traditional surgery.

## Data Availability Statement

The datasets presented in this study can be found in online repositories. The names of the repository/repositories and accession number(s) can be found below: The datasets analyzed in this study are collected from SEER repository (https://seer.cancer.gov/).

## Author Contributions

HY, Y-FW, and X-JC participated in the design of this project, interpretation of data, and drafting and critical revision of the article and provided final approval of the version to be submitted. HY and PC completed the data collection and analysis. All authors contributed to the article and approved the submitted version.

## Funding

This study was supported by the Medical Scientific Research Foundation of Zhejiang Province, China (Grant No. 2021KY1010). Key Laboratory of Diagnosis and Treatment of Digestive System Tumors of Zhejiang Province (2019E10020), the Ningbo Clinical Research Center for Digestive System Tumors (Grant No. 2019A21003), and the HwaMei Research Foundation of Ningbo No.2 Hosptial, China (Grant No. 2020HMKY55). Health Young Technical Backbone Talents Foundation of Ningbo.

## Conflict of Interest

The authors declare that the research was conducted in the absence of any commercial or financial relationships that could be construed as a potential conflict of interest.

## Publisher’s Note

All claims expressed in this article are solely those of the authors and do not necessarily represent those of their affiliated organizations, or those of the publisher, the editors and the reviewers. Any product that may be evaluated in this article, or claim that may be made by its manufacturer, is not guaranteed or endorsed by the publisher.

## References

[1] Buas MF Vaughan TL. Epidemiology and Risk Factors for Gastroesophageal Junction Tumors: Understanding the Rising Incidence of This Disease. Semin Radiat Oncol (2013) 23:3–9. doi: 10.1016/j.semradonc.2012.09.008 23207041 PMC3535292

[2] Kauppila JH Lagergren J. The Surgical Management of Esophago-Gastric Junctional Cancer. Surg Oncol (2016) 25:394–400. doi: 10.1016/j.suronc.2016.09.004 27916171

[3] Derakhshan MH Malekzadeh R Watabe H Yazdanbod A Fyfe V Kazemi A. Combination of Gastric Atrophy, Reflux Symptoms and Histological Subtype Indicates Two Distinct Aetiologies of Gastric Cardia Cancer. Gut (2008) 57:298–305. doi: 10.1136/gut.2007.137364 17965056

[4] Toyonaga T Man-i M East JE Nishino E Ono W Hirooka T. 1,635 Endoscopic Submucosal Dissection Cases in the Esophagus, Stomach, and Colorectum: Complication Rates and Long-Term Outcomes. Surg Endosc (2013) 27:1000–8. doi: 10.1007/s00464-012-2555-2 PMC3572381 23052530

[5] Kuwano H Nishimura Y Oyama T Kato H Kitagawa Y Kusano M. Guidelines for Diagnosis and Treatment of Carcinoma of the Esophagus April 2012 Edited by the Japan Esophageal Society. Esophagus Off J Jpn Esophageal Soc (2015) 12:1–30. doi: 10.1007/s10388-014-0465-1 PMC4297610 25620903

[6] Japanese Gastric Cancer Association. Japanese Gastric Cancer Treatment Guidelines 2018 (5th Edition). Gastric Cancer Off J Int Gastric Cancer Assoc Jpn Gastric Cancer Assoc (2020) 24:1–21. doi: 10.1007/s10120-020-01042-y PMC7790804 32060757

[7] Wingo PA Jamison PM Hiatt RA Weir HK Gargiullo PM Hutton M. Building the Infrastructure for Nationwide Cancer Surveillance and Control–a Comparison Between the National Program of Cancer Registries (NPCR) and the Surveillance, Epidemiology, and End Results (SEER) Program (United States). Cancer Causes Control CCC (2003) 14:175–93. doi: 10.1023/A:1023002322935 12749723

[8] Rice TW Ishwaran H Ferguson MK Blackstone EH Goldstraw P. Cancer of the Esophagus and Esophagogastric Junction: An Eighth Edition Staging Primer. J Thorac Oncol Off Publ Int Assoc Study Lung Cancer (2017) 12:36–42. doi: 10.1016/j.jtho.2016.10.016 PMC5591443 27810391

[9] Biondi A D’Ugo D Cananzi FC Papa V Borasi A Sicoli F. Does a Minimum Number of 16 Retrieved Nodes Affect Survival in Curatively Resected Gastric Cancer? Eur J Surg Oncol J Eur Soc Surg Oncol Br Assoc Surg Oncol (2015) 41:779–86. doi: 10.1016/j.ejso.2015.03.227 25899981

[10] In H Solsky I Palis B Langdon-Embry M Ajani J Sano T. Validation of the 8th Edition of the AJCC TNM Staging System for Gastric Cancer Using the National Cancer Database. Ann Surg Oncol (2017) 24:3683–91. doi: 10.1245/s10434-017-6078-x 28895113

[11] Hasegawa S Yoshikawa T Aoyama T Hayashi T Yamada T Tsuchida K. Esophagus or Stomach? The Seventh TNM Classification for Siewert Type II/III Junctional Adenocarcinoma. Ann Surg Oncol (2013) 20:773–9. doi: 10.1245/s10434-012-2780-x 23224137

[12] Ichihara S Uedo N Gotoda T. Considering the Esophagogastric Junction as a ‘Zone’. Dig Endosc Off J Jpn Gastroenterological Endosc Soc (2017) 29(Suppl 2):3–10. doi: 10.1111/den.12792 28425656

[13] Haverkamp L Ruurda JP van Leeuwen MS Siersema PD van Hillegersberg R. Systematic Review of the Surgical Strategies of Adenocarcinomas of the Gastroesophageal Junction. Surg Oncol (2014) 23:222–8. doi: 10.1016/j.suronc.2014.10.004 25466852

[14] Yoshinaga S Gotoda T Kusano C Oda I Nakamura K Takayanagi R. Clinical Impact of Endoscopic Submucosal Dissection for Superficial Adenocarcinoma Located at the Esophagogastric Junction. Gastrointestinal Endosc (2008) 67:202–9. doi: 10.1016/j.gie.2007.09.054 18226681

[15] Gertler R Stein HJ Schuster T Rondak IC Höfler H Feith M. Prevalence and Topography of Lymph Node Metastases in Early Esophageal and Gastric Cancer. Ann Surg (2014) 259:96–101. doi: 10.1097/SLA.0000000000000239 24096772

[16] Stein HJ Feith M Mueller J Werner M Siewert JR. Limited Resection for Early Adenocarcinoma in Barrett’s Esophagus. Ann Surg (2000) 232:733–42. doi: 10.1097/00000658-200012000-00002 PMC1421266 11088068

[17] Koufuji K Shirouzu K Aoyagi K Yano S Miyagi M Imaizumi T. Surgery and Clinicopathological Features of Gastric Adenocarcinoma Involving the Esophago-Gastric Junction. Kurume Med J (2005) 52:73–9. doi: 10.2739/kurumemedj.52.73 16422172

[18] Zhu M Cao B Li X Li P Wen Z Ji J. Risk Factors and a Predictive Nomogram for Lymph Node Metastasis of Superficial Esophagogastric Junction Cancer. J Gastroenterol Hepatol (2020) 35(9):1524–31. doi: 10.1111/jgh.15004 32023349

[19] Dubecz A Kern M Solymosi N Schweigert M Stein HJ. Predictors of Lymph Node Metastasis in Surgically Resected T1 Esophageal Cancer. Ann Thorac Surg (2015) 99:1879–85; discussion 1886. doi: 10.1016/j.athoracsur.2015.02.112 25929888

[20] Wani S Drahos J Cook MB Rastogi A Bansal A Yen R. Comparison of Endoscopic Therapies and Surgical Resection in Patients With Early Esophageal Cancer: A Population-Based Study. Gastrointestinal Endosc (2014) 79:224–32.e221. doi: 10.1016/j.gie.2013.08.002 PMC4042678 24060519

[21] Njei B McCarty TR Birk JW. Trends in Esophageal Cancer Survival in United States Adults From 1973 to 2009: A SEER Database Analysis. J Gastroenterol Hepatol (2016) 31:1141–6. doi: 10.1111/jgh.13289 PMC4885788 26749521

[22] Ngamruengphong S Wolfsen HC Wallace MB. Survival of Patients With Superficial Esophageal Adenocarcinoma After Endoscopic Treatment vs Surgery. Clin Gastroenterol Hepatol Off Clin Pract J Am Gastroenterological Assoc (2013) 11:1424–9.e1422; quiz e1481. doi: 10.1016/j.cgh.2013.05.025 PMC3889479 23735443

[23] Pyo JH Lee H Min YW Min BH Lee JH Kim KM. Indication for Endoscopic Treatment Based on the Risk of Lymph Node Metastasis in Patients With Siewert Type II/III Early Gastric Cancer. Gastric Cancer Off J Int Gastric Cancer Assoc Jpn Gastric Cancer Assoc (2018) 21:672–9. doi: 10.1007/s10120-017-0789-3 29243195

[24] Lagergren J Andersson G Talbäck M Drefahl S Bihagen E Härkönen J. Marital Status, Education, and Income in Relation to the Risk of Esophageal and Gastric Cancer by Histological Type and Site. Cancer (2016) 122:207–12. doi: 10.1002/cncr.29731 26447737

[25] Pouw RE Heldoorn N Alvarez Herrero L ten Kate FJ Visser M Busch OR. Do We Still Need EUS in the Workup of Patients With Early Esophageal Neoplasia? A Retrospective Analysis of 131 Cases. Gastrointestinal Endosc (2011) 73:662–8. doi: 10.1016/j.gie.2010.10.046 21272876

